# (*Z*)-Methyl 2-[(4-bromo-2-formyl­phen­oxy)meth­yl]-3-*o*-tolyl­acrylate

**DOI:** 10.1107/S1600536811037731

**Published:** 2011-09-20

**Authors:** S. Vijayakumar, R. Madhanraj, S. Murugavel, R. Selvakumar, M. Bakthadoss

**Affiliations:** a Department of Physics, Sri Balaji Chokkalingam Engineering College, Arni, Thiruvannamalai 632 317, India; b Department of Physics, Ranipettai Engineering College, Thenkadapathangal, Walaja 632 513, India; c Department of Physics, Thanthai Periyar Government Institute of Technology, Vellore 632 002, India; d Department of Organic Chemistry, University of Madras, Maraimalai Campus, Chennai 600 025, India

## Abstract

In the title compound, C_19_H_17_BrO_4_, the dihedral angle between the two benzene rings is 82.1 (1)°. The mol­ecular structure is stabilized by an intra­molecular C—H⋯O hydrogen bond which generates an *S*(7) ring motif. The crystal packing is stabilized by inter­molecular C—H⋯O hydrogen bonds and C—H⋯π inter­actions. Inter­molecular C—H⋯O inter­actions are involved in the formation of centrosymmetric *R*_2_^2^(16) dimers, which are connected into supra­molecular tapes running along the [100] direction.

## Related literature

For background to the applications of acrylates, see: de Fraine *et al.* (1991 ▶); Zhang & Ji (1992 ▶). For related structures, see: Wang *et al.* (2011 ▶); Hou (2008 ▶). For hydrogen-bond motifs, see: Bernstein *et al.* (1995 ▶).
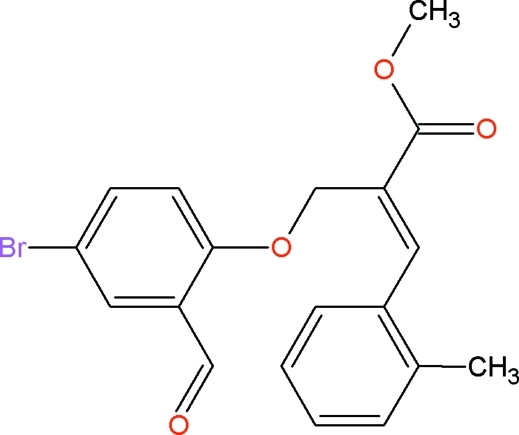


## Experimental

### 

#### Crystal data

C_19_H_17_BrO_4_
*M* *_r_* = 389.24
Triclinic, 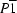

*a* = 8.0114 (2) Å
*b* = 8.6138 (2) Å
*c* = 13.4827 (4) Å
α = 96.466 (1)°
β = 97.185 (1)°
γ = 106.546 (2)°
*V* = 874.08 (4) Å^3^
*Z* = 2
Mo *K*α radiation
μ = 2.37 mm^−1^
*T* = 293 K
0.25 × 0.23 × 0.18 mm

#### Data collection

Bruker APEXII CCD diffractometer
Absorption correction: multi-scan (*SADABS*; Sheldrick, 1996 ▶) *T*_min_ = 0.547, *T*_max_ = 0.653
21788 measured reflections
5440 independent reflections
2870 reflections with *I* > 2σ(*I*)
*R*_int_ = 0.025

#### Refinement

*R*[*F* ^2^ > 2σ(*F* ^2^)] = 0.043
*wR*(*F* ^2^) = 0.125
*S* = 1.02
5440 reflections
219 parameters
H-atom parameters constrained
Δρ_max_ = 0.61 e Å^−3^
Δρ_min_ = −0.61 e Å^−3^

### 

Data collection: *APEX2* (Bruker, 2004 ▶); cell refinement: *APEX2* and *SAINT* (Bruker, 2004 ▶); data reduction: *SAINT* and *XPREP* (Bruker, 2004 ▶); program(s) used to solve structure: *SHELXS97* (Sheldrick, 2008 ▶); program(s) used to refine structure: *SHELXL97* (Sheldrick, 2008 ▶); molecular graphics: *ORTEP-3* (Farrugia, 1997 ▶); software used to prepare material for publication: *SHELXL97* and *PLATON* (Spek, 2009 ▶).

## Supplementary Material

Crystal structure: contains datablock(s) global, I. DOI: 10.1107/S1600536811037731/bt5640sup1.cif

Structure factors: contains datablock(s) I. DOI: 10.1107/S1600536811037731/bt5640Isup2.hkl

Supplementary material file. DOI: 10.1107/S1600536811037731/bt5640Isup3.cml

Additional supplementary materials:  crystallographic information; 3D view; checkCIF report

## Figures and Tables

**Table 1:** Hydrogen-bond geometry (Å, °) *Cg* is the centroid of the C13–C18 benzene ring.

*D*—H⋯*A*	*D*—H	H⋯*A*	*D*⋯*A*	*D*—H⋯*A*
C14—H14⋯O3	0.93	2.59	3.377 (3)	143
C19—H19*B*⋯O1^i^	0.96	2.53	3.436 (3)	157
C5—H5⋯O4^ii^	0.93	2.44	3.273 (3)	149
C19—H19*C*⋯*Cg*^iii^	0.96	2.74	3.580 (3)	147

Symmetry codes: (i) 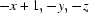; (ii) 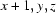; (iii) 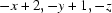.

**Fig. 1.**
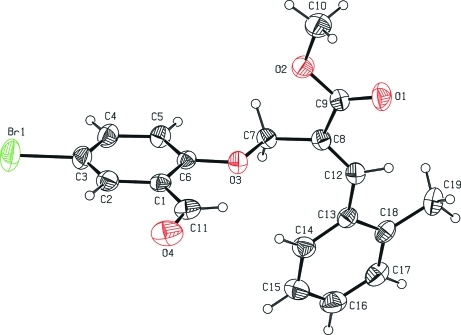
The molecular structure of the title compound with the atom numbering scheme. Displacement ellipsoids are drawn at the 30% probability level. H atoms are presented as a small cycles of arbitrary radius.

**Fig. 2.**
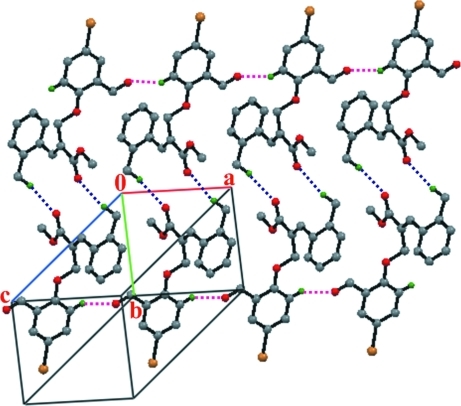
Supramolecular tape formation in the crystal packing of the title compound whereby centrosymmetrc R22(16) dimeric aggregates sustained by C—H···O (blue dashed lines) contacts are linked via C—H···O contacts (magenta dashed lines) along [1 0 0].

**Fig. 3.**
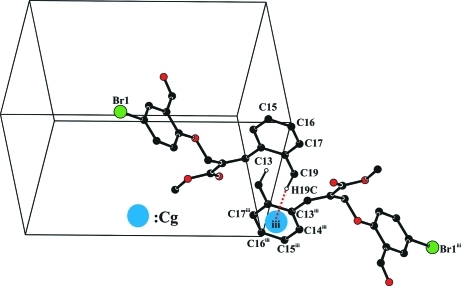
A view of the C-H···π interactions (dotted lines) in the crystal structure of the title compound. Cg denotes centroid of the C13-C18 benzene ring. [Symmetry code: (iii) 2-x, 1-y, -z.]

## supplementary crystallographic information

### Comment

Acrylate and its derivatives are important compounds because of their agrochemical and medical applications (de Fraine *et al.,* 1991; Zhang & Ji, 1992).

Fig. 1. shows a displacement ellipsoid plot of the title compound with the atom numbering scheme. The dihedral angle between the two aromatic rings is 82.1 (1)°. The methyl acrylate (O1/O2/C7-C10) plane forms dihedral angles of 84.9 (1)° and 41.5 (1)°, respectively, with the bromo formyl phenyl and methyl phenyl rings. The geometric parameters of the title molecule agrees well with those reported for similar structures (Wang *et al.*, 2011; Hou, 2008).

The molecular structure is stabilized by intramolecular C14—H14···O3 hydrogen bond which generates an S(7) ring motif. The crystal packing is stabilized by intermolecular C—H···O hydrogen bonds. The molecules at *x, y, z* and *1-x, -y, -z* are linked by C19—H19B···O1 hydrogen bonds into cyclic centrosymmetric *R_2_^2^*(16) dimers. The dimers are linked by the C5—H5···O4 hydrogen bond forming supramolecular tapes running along the [100] directions (Fig. 2). The crystal packing is further stabilized by C—H···π interactions between a methyl H19C atom and a neighbouring benzene ring (C13-C18), with a C19—H19C···Cg^iii^ separation of 2.74 Å ( Fig. 3 and Table 1; Cg is the centroid of the C13-C18 benzene ring, Symmetry code as in Fig. 3).

### Experimental

A solution of salicylaldehyde (3.7 mmol, 0.74g) and potassium carbonate (5.59 mmol, 0.77g) in acetonitrile as solvent (10ml) was stirred for 15 minutes at room temperature. To this solution, (Z-methyl 2-(bromomethyl)-3-o-tolylacrylate (3.7 mmol, 1g) was added dropwise. After the completion of the reaction as indicated by TLC, acetonitrile was evaporated. Ethylacetate (15ml) and water (15ml) were added to the crude mass and extracted. The organic layer was dried over anhydrous sodium sulfate. Removal of the solvent led to the crude product which was purified through pad of silica gel (100-200 mesh) using ethylacetate and hexanes (1:9) as solvents. The pure title compound was obtained as a colorless solid (1.32g, 91%). Single crystals suitable for X-ray diffraction were obtained by slow evaporation of a ethylacetate solution at room temperature.

### Refinement

All   H atoms were positioned geometrically, with C-H = 0.93 - 0.96 Å and constrained to ride on their parent atom, with *U*_iso_(H)=1.5*U*_eq_ for methyl and hydroxyl H atoms and 1.2*U*_eq_(C) for other H atoms.

### Crystal data

**Table:**

C_19_H_17_BrO_4_	*Z* = 2
*M**_r_* = 389.24	*F*(000) = 396
Triclinic, *P*1	*D*_x_ = 1.479 Mg m^−^^3^
Hall symbol: -P 1	Mo *K*α radiation, λ = 0.71073 Å
*a* = 8.0114 (2) Å	Cell parameters from 5491 reflections
*b* = 8.6138 (2) Å	θ = 1.5–30.8°
*c* = 13.4827 (4) Å	µ = 2.37 mm^−^^1^
α = 96.466 (1)°	*T* = 293 K
β = 97.185 (1)°	Block, colourless
γ = 106.546 (2)°	0.25 × 0.23 × 0.18 mm
*V* = 874.08 (4) Å^3^	

### Data collection

**Table:**

Bruker APEXII CCD diffractometer	5440 independent reflections
Radiation source: fine-focus sealed tube	2870 reflections with *I* > 2σ(*I*)
graphite	*R*_int_ = 0.025
Detector resolution: 10.0 pixels mm^-1^	θ_max_ = 30.8°, θ_min_ = 2.5°
ω scans	*h* = −11→8
Absorption correction: multi-scan (*SADABS*; Sheldrick, 1996)	*k* = −9→12
*T*_min_ = 0.547, *T*_max_ = 0.653	*l* = −19→19
21788 measured reflections	

### Refinement

**Table:**

Refinement on *F*^2^	Primary atom site location: structure-invariant direct methods
Least-squares matrix: full	Secondary atom site location: difference Fourier map
*R*[*F*^2^ > 2σ(*F*^2^)] = 0.043	Hydrogen site location: inferred from neighbouring sites
*wR*(*F*^2^) = 0.125	H-atom parameters constrained
*S* = 1.02	*w* = 1/[σ^2^(*F*_o_^2^) + (0.0517*P*)^2^ + 0.2207*P*] where *P* = (*F*_o_^2^ + 2*F*_c_^2^)/3
5440 reflections	(Δ/σ)_max_ < 0.001
219 parameters	Δρ_max_ = 0.61 e Å^−^^3^
0 restraints	Δρ_min_ = −0.61 e Å^−^^3^

### Special details

**Table:**

Geometry. All esds (except the esd in the dihedral angle between two l.s. planes) are estimated using the full covariance matrix. The cell esds are taken into account individually in the estimation of esds in distances, angles and torsion angles; correlations between esds in cell parameters are only used when they are defined by crystal symmetry. An approximate (isotropic) treatment of cell esds is used for estimating esds involving l.s. planes.
Refinement. Refinement of F^2^ against ALL reflections. The weighted R-factor wR and goodness of fit S are based on F^2^, conventional R-factors R are based on F, with F set to zero for negative F^2^. The threshold expression of F^2^ > 2sigma(F^2^) is used only for calculating R-factors(gt) etc. and is not relevant to the choice of reflections for refinement. R-factors based on F^2^ are statistically about twice as large as those based on F, and R- factors based on ALL data will be even larger.

### Fractional atomic coordinates and isotropic or equivalent isotropic displacement parameters (Å^2^)

**Table:**

	*x*	*y*	*z*	*U*_iso_*/*U*_eq_	
C1	0.5938 (3)	0.6570 (2)	0.44131 (14)	0.0451 (4)	
C2	0.6433 (3)	0.7625 (3)	0.53364 (16)	0.0582 (6)	
H2	0.5581	0.7944	0.5644	0.070*	
C3	0.8167 (4)	0.8193 (3)	0.57901 (16)	0.0628 (6)	
C4	0.9439 (3)	0.7725 (3)	0.53421 (17)	0.0620 (6)	
H4	1.0614	0.8124	0.5654	0.074*	
C5	0.8989 (3)	0.6674 (3)	0.44374 (16)	0.0522 (5)	
H5	0.9853	0.6360	0.4140	0.063*	
C6	0.7237 (2)	0.6087 (2)	0.39719 (14)	0.0419 (4)	
C7	0.7942 (2)	0.4478 (2)	0.26070 (15)	0.0460 (4)	
H7A	0.8629	0.4046	0.3086	0.055*	
H7B	0.8741	0.5375	0.2364	0.055*	
C8	0.6943 (3)	0.3164 (2)	0.17415 (15)	0.0462 (4)	
C9	0.6192 (3)	0.1482 (3)	0.19567 (16)	0.0514 (5)	
C10	0.5959 (4)	−0.0215 (3)	0.3216 (2)	0.0829 (8)	
H10A	0.4703	−0.0646	0.3009	0.124*	
H10B	0.6242	−0.0147	0.3936	0.124*	
H10C	0.6521	−0.0925	0.2885	0.124*	
C11	0.4093 (3)	0.6015 (3)	0.39152 (18)	0.0588 (6)	
H11	0.3784	0.5237	0.3333	0.071*	
C12	0.6729 (3)	0.3375 (3)	0.07702 (15)	0.0494 (5)	
H12	0.6170	0.2431	0.0304	0.059*	
C13	0.7263 (2)	0.4903 (3)	0.03471 (15)	0.0490 (5)	
C14	0.7153 (3)	0.6369 (3)	0.08434 (18)	0.0598 (5)	
H14	0.6751	0.6385	0.1461	0.072*	
C15	0.7630 (4)	0.7799 (3)	0.0434 (2)	0.0711 (7)	
H15	0.7554	0.8770	0.0775	0.085*	
C16	0.8218 (4)	0.7776 (3)	−0.0476 (2)	0.0743 (7)	
H16	0.8566	0.8742	−0.0747	0.089*	
C17	0.8297 (3)	0.6337 (3)	−0.09913 (18)	0.0651 (6)	
H17	0.8673	0.6340	−0.1617	0.078*	
C18	0.7828 (3)	0.4878 (3)	−0.06005 (15)	0.0531 (5)	
C19	0.7942 (3)	0.3325 (3)	−0.11804 (17)	0.0655 (6)	
H19A	0.8274	0.3532	−0.1824	0.098*	
H19B	0.6814	0.2499	−0.1283	0.098*	
H19C	0.8810	0.2951	−0.0804	0.098*	
O1	0.5339 (3)	0.0321 (2)	0.13521 (13)	0.0794 (5)	
O2	0.6575 (2)	0.13951 (18)	0.29434 (12)	0.0652 (4)	
O3	0.66638 (17)	0.50451 (17)	0.30849 (10)	0.0497 (3)	
O4	0.2955 (2)	0.6501 (3)	0.42096 (16)	0.0877 (6)	
Br1	0.88459 (5)	0.96282 (4)	0.70416 (2)	0.10598 (18)	

### Atomic displacement parameters (Å^2^)

**Table:**

	*U*^11^	*U*^22^	*U*^33^	*U*^12^	*U*^13^	*U*^23^
C1	0.0500 (11)	0.0487 (11)	0.0443 (10)	0.0191 (9)	0.0157 (9)	0.0186 (8)
C2	0.0751 (16)	0.0628 (13)	0.0495 (11)	0.0316 (12)	0.0236 (11)	0.0185 (10)
C3	0.0861 (18)	0.0606 (13)	0.0405 (10)	0.0204 (12)	0.0117 (11)	0.0069 (9)
C4	0.0599 (13)	0.0636 (14)	0.0521 (12)	0.0087 (11)	−0.0021 (10)	0.0051 (10)
C5	0.0449 (11)	0.0578 (12)	0.0510 (11)	0.0123 (10)	0.0073 (9)	0.0060 (9)
C6	0.0445 (10)	0.0420 (10)	0.0391 (9)	0.0107 (8)	0.0080 (8)	0.0116 (8)
C7	0.0394 (10)	0.0467 (10)	0.0528 (11)	0.0139 (8)	0.0105 (8)	0.0051 (8)
C8	0.0423 (10)	0.0476 (11)	0.0491 (11)	0.0139 (9)	0.0104 (8)	0.0048 (8)
C9	0.0536 (12)	0.0490 (11)	0.0504 (11)	0.0142 (10)	0.0107 (9)	0.0035 (9)
C10	0.106 (2)	0.0545 (14)	0.0797 (18)	0.0085 (14)	0.0066 (15)	0.0254 (13)
C11	0.0498 (12)	0.0710 (14)	0.0669 (14)	0.0253 (11)	0.0187 (11)	0.0282 (12)
C12	0.0434 (10)	0.0529 (12)	0.0499 (11)	0.0135 (9)	0.0078 (9)	0.0027 (9)
C13	0.0396 (10)	0.0567 (12)	0.0482 (11)	0.0134 (9)	0.0021 (8)	0.0073 (9)
C14	0.0595 (13)	0.0647 (14)	0.0619 (13)	0.0268 (11)	0.0123 (11)	0.0137 (11)
C15	0.0777 (16)	0.0630 (15)	0.0803 (18)	0.0342 (13)	0.0083 (14)	0.0139 (13)
C16	0.0793 (17)	0.0678 (16)	0.0757 (17)	0.0188 (14)	0.0051 (14)	0.0284 (14)
C17	0.0607 (14)	0.0744 (16)	0.0530 (13)	0.0085 (12)	0.0033 (10)	0.0187 (12)
C18	0.0398 (10)	0.0650 (13)	0.0457 (11)	0.0067 (10)	−0.0025 (8)	0.0072 (10)
C19	0.0609 (14)	0.0723 (15)	0.0493 (12)	0.0043 (12)	0.0074 (10)	−0.0049 (11)
O1	0.1097 (14)	0.0511 (9)	0.0574 (9)	0.0001 (9)	0.0075 (9)	−0.0037 (8)
O2	0.0781 (11)	0.0482 (9)	0.0593 (9)	0.0066 (8)	−0.0001 (8)	0.0129 (7)
O3	0.0386 (7)	0.0575 (8)	0.0496 (8)	0.0131 (6)	0.0068 (6)	−0.0009 (6)
O4	0.0632 (11)	0.1185 (16)	0.1058 (15)	0.0513 (11)	0.0325 (10)	0.0350 (12)
Br1	0.1485 (4)	0.1068 (3)	0.05066 (17)	0.0334 (2)	0.00845 (17)	−0.01403 (15)

### Geometric parameters (Å, °)

**Table:**

C1—C2	1.396 (3)	C10—H10A	0.9600
C1—C6	1.398 (3)	C10—H10B	0.9600
C1—C11	1.464 (3)	C10—H10C	0.9600
C2—C3	1.370 (3)	C11—O4	1.197 (3)
C2—H2	0.9300	C11—H11	0.9300
C3—C4	1.378 (3)	C12—C13	1.466 (3)
C3—Br1	1.895 (2)	C12—H12	0.9300
C4—C5	1.376 (3)	C13—C14	1.392 (3)
C4—H4	0.9300	C13—C18	1.408 (3)
C5—C6	1.387 (3)	C14—C15	1.380 (3)
C5—H5	0.9300	C14—H14	0.9300
C6—O3	1.354 (2)	C15—C16	1.369 (4)
C7—O3	1.443 (2)	C15—H15	0.9300
C7—C8	1.496 (3)	C16—C17	1.373 (4)
C7—H7A	0.9700	C16—H16	0.9300
C7—H7B	0.9700	C17—C18	1.389 (3)
C8—C12	1.339 (3)	C17—H17	0.9300
C8—C9	1.478 (3)	C18—C19	1.504 (3)
C9—O1	1.191 (3)	C19—H19A	0.9600
C9—O2	1.343 (3)	C19—H19B	0.9600
C10—O2	1.440 (3)	C19—H19C	0.9600
			
C2—C1—C6	118.86 (19)	H10A—C10—H10C	109.5
C2—C1—C11	119.93 (19)	H10B—C10—H10C	109.5
C6—C1—C11	121.20 (19)	O4—C11—C1	124.1 (2)
C3—C2—C1	120.2 (2)	O4—C11—H11	117.9
C3—C2—H2	119.9	C1—C11—H11	117.9
C1—C2—H2	119.9	C8—C12—C13	128.39 (19)
C2—C3—C4	120.4 (2)	C8—C12—H12	115.8
C2—C3—Br1	120.44 (18)	C13—C12—H12	115.8
C4—C3—Br1	119.17 (19)	C14—C13—C18	119.1 (2)
C5—C4—C3	120.6 (2)	C14—C13—C12	121.34 (19)
C5—C4—H4	119.7	C18—C13—C12	119.46 (19)
C3—C4—H4	119.7	C15—C14—C13	121.1 (2)
C4—C5—C6	119.5 (2)	C15—C14—H14	119.4
C4—C5—H5	120.2	C13—C14—H14	119.4
C6—C5—H5	120.2	C16—C15—C14	119.5 (2)
O3—C6—C5	123.87 (17)	C16—C15—H15	120.2
O3—C6—C1	115.80 (17)	C14—C15—H15	120.2
C5—C6—C1	120.33 (18)	C15—C16—C17	120.4 (2)
O3—C7—C8	107.34 (15)	C15—C16—H16	119.8
O3—C7—H7A	110.2	C17—C16—H16	119.8
C8—C7—H7A	110.2	C16—C17—C18	121.5 (2)
O3—C7—H7B	110.2	C16—C17—H17	119.2
C8—C7—H7B	110.2	C18—C17—H17	119.2
H7A—C7—H7B	108.5	C17—C18—C13	118.2 (2)
C12—C8—C9	116.79 (19)	C17—C18—C19	120.2 (2)
C12—C8—C7	124.91 (19)	C13—C18—C19	121.5 (2)
C9—C8—C7	118.25 (18)	C18—C19—H19A	109.5
O1—C9—O2	122.5 (2)	C18—C19—H19B	109.5
O1—C9—C8	125.9 (2)	H19A—C19—H19B	109.5
O2—C9—C8	111.64 (18)	C18—C19—H19C	109.5
O2—C10—H10A	109.5	H19A—C19—H19C	109.5
O2—C10—H10B	109.5	H19B—C19—H19C	109.5
H10A—C10—H10B	109.5	C9—O2—C10	115.46 (19)
O2—C10—H10C	109.5	C6—O3—C7	118.17 (15)
			
C6—C1—C2—C3	1.0 (3)	C9—C8—C12—C13	177.09 (19)
C11—C1—C2—C3	−177.82 (19)	C7—C8—C12—C13	−5.6 (3)
C1—C2—C3—C4	−0.2 (3)	C8—C12—C13—C14	−37.2 (3)
C1—C2—C3—Br1	179.89 (15)	C8—C12—C13—C18	145.6 (2)
C2—C3—C4—C5	−0.5 (4)	C18—C13—C14—C15	−1.7 (3)
Br1—C3—C4—C5	179.47 (17)	C12—C13—C14—C15	−179.0 (2)
C3—C4—C5—C6	0.3 (3)	C13—C14—C15—C16	0.2 (4)
C4—C5—C6—O3	−179.72 (19)	C14—C15—C16—C17	1.4 (4)
C4—C5—C6—C1	0.5 (3)	C15—C16—C17—C18	−1.5 (4)
C2—C1—C6—O3	179.09 (17)	C16—C17—C18—C13	0.0 (3)
C11—C1—C6—O3	−2.1 (3)	C16—C17—C18—C19	−179.6 (2)
C2—C1—C6—C5	−1.2 (3)	C14—C13—C18—C17	1.5 (3)
C11—C1—C6—C5	177.62 (19)	C12—C13—C18—C17	178.89 (19)
O3—C7—C8—C12	100.2 (2)	C14—C13—C18—C19	−178.89 (19)
O3—C7—C8—C9	−82.5 (2)	C12—C13—C18—C19	−1.5 (3)
C12—C8—C9—O1	−4.1 (3)	O1—C9—O2—C10	2.9 (3)
C7—C8—C9—O1	178.4 (2)	C8—C9—O2—C10	−177.5 (2)
C12—C8—C9—O2	176.27 (18)	C5—C6—O3—C7	1.6 (3)
C7—C8—C9—O2	−1.2 (3)	C1—C6—O3—C7	−178.66 (16)
C2—C1—C11—O4	5.7 (3)	C8—C7—O3—C6	171.39 (15)
C6—C1—C11—O4	−173.0 (2)		

### Hydrogen-bond geometry (Å, °)

**Table:**

*D*—H···*A*	*D*—H	H···*A*	*D*···*A*	*D*—H···*A*
C14—H14···O3	0.93	2.59	3.377 (3)	143
C19—H19B···O1^i^	0.96	2.53	3.436 (3)	157
C5—H5···O4^ii^	0.93	2.44	3.273 (3)	149
C19—H19C···Cg^iii^	0.96	2.74	3.580 (3)	147

Symmetry codes:  (i) −*x*+1, −*y*, −*z*; (ii) *x*+1, *y*, *z*; (iii) −*x*+2, −*y*+1, −*z*.
